# A sneak preview

**DOI:** 10.1007/s12471-021-01623-1

**Published:** 2021-09-09

**Authors:** A. W. G. J. Oomen

**Affiliations:** Department of Cardiology, Diakonessenhuis, Utrecht, The Netherlands

A 67-year-old female was seen at the outpatient clinic because of recurrent palpitations. She had no relevant past medical history. Physical examination and echocardiography did not reveal any abnormalities. An electrocardiogram (ECG) was recorded (Fig. 1).

**Fig. 1:**
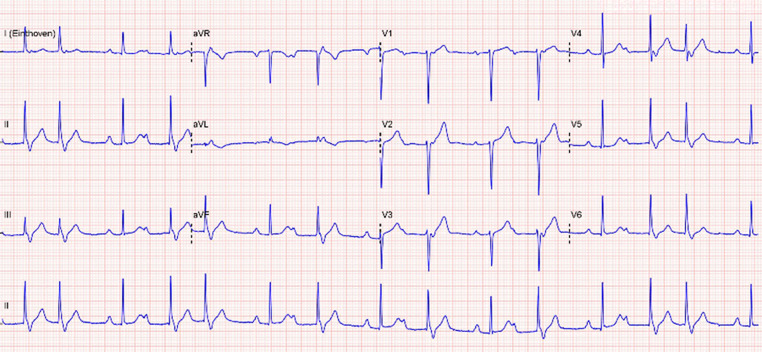
Electrocardiogram at presentation

How do you explain the rhythm in Fig. 1, and what arrhythmia is likely to be the cause of her palpitations?

## Answer

You will find the answer elsewhere in this issue.

